# Interaction of repetition and retention interval influences the transfer effect after answer feedback for episodic memory

**DOI:** 10.3389/fpsyg.2025.1638780

**Published:** 2025-11-03

**Authors:** Lingwei Wang, Jiongjiong Yang

**Affiliations:** School of Psychological and Cognitive Sciences and Beijing Key Laboratory of Behavior and Mental Health, Peking University, Beijing, China

**Keywords:** transfer effect, answer feedback, repetition, retention interval, episodic memory

## Abstract

**Introduction:**

Retrieval practice with answer feedback is an efficient way to enhance episodic memory, but previous studies fail to find a robust transfer of learning for non-trained information. The aim of this study was to clarify the boundary conditions for the transfer effect after answer feedback.

**Methods:**

Two groups of participants learned episodic sentences through single or repeated study and training (ST, SSTT), then they were tested at 10 min, 1 day and 1 week. During the training phase, only half of the items were trained under conditions of feedback, no feedback, or restudy, while the other half items were not trained.

**Results:**

The transfer effect (i.e., feedback vs. restudy condition for the non-trained items) was influenced by the interaction of repetition and retention interval, as it appeared at 10 min after SSTT but not after ST. Moreover, the transfer effect declined over time, and was significantly lower than chance level at 1 week after SSTT.

**Discussion:**

The results suggest that the transfer effect after answer feedback could be obtained after repeated study and training for the episodic information, but it is short-lived. They also highlight the time change of memory specificity and generalization due to answer feedback.

## Introduction

Episodic memory is an important cognitive function, by which people remember various events with contextual information. How to effectively enhance episodic memory is a critical issue in memory research and educational practice. One available strategy is retrieval practice with answer feedback (e.g., classroom quizzes or tests followed with correct answer) (Pashler et al., 2007; Roediger and Butler, 2011; Dunlosky et al., 2013; Butler and Woodward, 2018). Compared with restudy strategy (e.g., rereading), retrieval practice with answer feedback can better enhance the memory performance. This memory benefit from retrieval practice (with or without feedback) on the trained information is referred to as the testing effect (Roediger and Karpicke, 2006; Roediger and Butler, 2011; Rowland, 2014). However, in a variety of circumstances, it is impractical to train and provide feedback on all to-be-learned materials (Pan and Rickard, 2018). This is especially the case for episodic events when only part of information is trained followed by answer feedback, and both the trained and non-trained materials share the same episode. Therefore, it is necessary to clarify whether answer feedback facilitates positive transfer of learning on non-trained episodic information.

Transfer of learning usually refers to as the use of prior learning in a new context (Carpenter, 2012). The context types substantially vary in transfer studies such as temporal context, test format and knowledge domain. In addition, the transfer of learning can be observed in the dimension of content (Barnett and Ceci, 2002; Pan and Rickard, 2018). Align with this idea, in the category of “untested materials seen during initial study” of Pan and Rickard (2018), the transfer effect is defined as memory difference between retrieval practice/feedback condition and restudy condition in how well participants remember the non-trained information (see also in Butler, 2010; McDaniel et al., 2012). In some studies, the memory difference for non-trained information is assessed by retrieval practice/feedback against control (e.g., no training experience after learning) rather than restudy condition, which is referred to as the retrieval-induced facilitation (e.g., Chan, 2009; Liu and Ranganath, 2021).

Previous studies have observed positive transfer effect (e.g., Jonker et al., 2018; Wang and Yang, 2023) or retrieval-induced facilitation (e.g., Chan, 2009; Liu and Ranganath, 2021) for non-trained episodic information (e.g., scene-object associations or episodic sentences). For example, in a study of Jonker et al. (2018), participants first learned 12 scene-object pairs, in which two back-to-back trials shared the same scene, and then they performed retrieval or restudy on one of the two objects paired with the same scene during the training phase. Result showed that the final recall performance at 24 h was enhanced for retrieval practice vs. restudy even when the objects were not trained (i.e., transfer effect). It suggests that retrieval of some episodic information would facilitate retention of the content-related non-retrieved information.

On the other hand, when answer feedback is provided after retrieval practice, most studies on the transfer effect focus on knowledge scope, including paragraphs and concepts from textbook (e.g., McDaniel et al., 2007; Butler, 2010; Wooldridge et al., 2014; Tran et al., 2015; Pan et al., 2016a; Eglington and Kang, 2018), term-definition facts (e.g., Pan and Rickard, 2017), and word triplets (e.g., Pan et al., 2016b). Few studies have explored the transfer effect due to answer feedback for episodic information (Pickering et al., 2021). Moreover, in most of semantic/knowledge-based studies, the positive transfer effect is not reliably observed when answer feedback is provided. As shown in the meta-analysis of Pan and Rickard (2018), the transfer effect in the category of “untested materials seen during initial study” is small when studies with and without answer feedback are included. Similarly, when episodic information is adopted (Pickering et al., 2021), participants learned multielement triplets and trained on one pairwise association from each triplet through retrieval practice with feedback or restudy condition. There was no evidence for a transfer effect compared to restudy at final test 2 days later, although non-trained pairs in both feedback and restudy conditions were better remembered than those without any training experience (i.e., control condition). Then, could answer feedback promote transfer of learning of episodic information? Whether there are boundary conditions for the reliable transfer effect due to answer feedback when compared with restudy condition?

To answer these questions, we focused on two important factors in this study. The first is the integration level between trained and non-trained items. Various theories on retrieval practice indicate that information integration is important for the transfer effect, whether the information is semantically (Carpenter, 2009; Pyc and Rawson, 2010) or episodically related (Karpicke et al., 2014). The integration can be improved through repeated study and repeated training (e.g., Jonker et al., 2018; Himmer et al., 2019; Wang and Yang, 2023). However, in many studies of transfer effect with answer feedback, even when the training is applied at least twice, participants usually have only one study opportunity, whether using episodic (Pickering et al., 2021), or semantic materials (e.g., Wooldridge et al., 2014; Tran et al., 2015; Pan et al., 2016a,b; Pan and Rickard, 2017). The positive transfer effect is usually observed in studies when participants study passages and textbooks with sufficient amount of time (e.g., McDaniel et al., 2007; Eglington and Kang, 2018). Recent findings have suggested that repeated study and repeated training are both necessary for the transfer effect, and applying a single study or a single training does not lead to a significant transfer effect (Jonker et al., 2018; Wang and Yang, 2023). Furthermore, repeated study and repeated training improve the initial performance (Karpicke and Roediger, 2008), which is positively related to the magnitude of the transfer effect (Pan and Rickard, 2018). Therefore, sufficient study and training repetitions should be both applied for the transfer effect with answer feedback.

The second important factor is the retention interval. One distinctive feature of answer feedback is its selectivity, by which memory of specific information with feedback is enhanced, and memory of other information that does not receive feedback is inhibited (e.g., Butler et al., 2008; Kornell et al., 2009, 2015). In contrast, retrieval practice facilitates memory integration of both trained and non-trained related information (Carpenter, 2009; Roediger and Butler, 2011; Karpicke et al., 2014; Antony et al., 2017) especially after sleep (e.g., Chan, 2009; Jonker et al., 2018; Liu and Ranganath, 2021). As answer feedback is provided after retrieval practice, the transfer effect induced by the information integration during training may be affected by subsequent memory selectivity over time. However, most previous studies have only used one interval (e.g., 2 days: Pan and Rickard, 2017; Pickering et al., 2021; 1 week and more: McDaniel et al., 2007; Pan et al., 2016b), and few studies have explored whether the transfer of learning due to answer feedback changes over time (Tran et al., 2015). Including three intervals enabled us to clarify this issue. Studies have suggested that retrieval attempt activates related knowledge, and subsequent answer feedback strengthens appropriate connections and weakens inappropriate connections (e.g., Kornell et al., 2015; Kornell and Vaughn, 2016; Butler and Woodward, 2018). Therefore, it is possible that memory of non-trained information is facilitated shortly after answer feedback if memory integration is enhanced through repeated study and training. Then the transfer effect would decrease over time as memory selectivity due to answer feedback is strengthened and leads to weakened non-trained information when compared with restudy or no-feedback condition.

To summarize, the objective of this study was to explore the boundary conditions under which answer feedback influenced the transfer effect for episodic information over time. Sentences that described two or three consecutive episodes were created. Two groups of participants studied episodic sentences by a single or repeated study (S) and training (T) (i.e., study once and training once as ST, study twice and training twice as SSTT). During the training phase, only half of the items were trained in three conditions (feedback, no-feedback and restudy) (i.e., trained items), and the other half items were not presented (i.e., non-trained items). After intervals of 10 min, 1 day and 1 week, the participants recalled all the items of the sentences and rated the confidence. To identify the effects of repeated study and training, the participants rated familiarity during study and vividness during both study and training phases.

The memory effects included the testing effect, transfer effect and feedback effect. When compared with restudy condition, the memory benefit from the feedback condition was defined as the testing effect and transfer effect for the trained item and non-trained items, respectively. When compared with no-feedback condition, the memory benefit from the feedback condition was defined as the feedback effect. In addition to memory accuracy, the confidence level, which reflected individuals' metacognition of their own memory abilities and vividness (Yonelinas, 1994; Chua et al., 2008; Dunlosky and Metcalfe, 2009), was also analyzed. As memory selectivity of answer feedback has different influence on the trained and non-trained items (i.e., item type) over time, we predicted that there would be an interaction of item type with group and retention interval for memory effects. Specifically, the testing effect would appear after both ST and SSTT and increase over time. Differently, the transfer effect would appear only after SSTT at 10 min, then it would decrease over time and even become negative after a longer interval when SSTT is applied. The feedback effect for the non-trained information would also be significant only at a shorter interval after SSTT. The confidence rating would show similar results to those of overall accuracy.

## Materials and methods

### Participants

The overall sample size was based on a prior power analysis using MorePower 6.0.4 (Campbell and Thompson, 2012). For the interaction between group (ST, SSTT), item/effect type (trained, non-trained) and retention interval (10 min, 1 day, 1 week) in the testing/transfer effect, a total sample of at least 60 participants would be required with an adequate power (α = 0.05, power = 0.80) to detect a medium to large effect size ($\eta_{p}^{2}$ = 0.08). The effect size was selected based on previous studies reporting a medium to large effect size ($\eta_{p}^{2}$ = 0.04–0.25) for the interaction about item type or retention interval (e.g., McDaniel et al., 2007; Eglington and Kang, 2018; Jonker et al., 2018; Wang and Yang, 2023) in the positive transfer effect. Thus, 72 participants (19 males and 53 females, with mean age of 21.74 ± 2.25 years) were recruited, with 36 participants in each group. The participants were recruited through the online notice on the Bulletin Board System of Peking University. All of the participants were native Chinese speakers, right-handed, and they all gave written informed consent in accordance with the procedures and protocols, which were approved by the Review Board of School of Psychological and Cognitive Sciences, Peking University.

### Design and materials

One between-subjects factor was manipulated: group (ST, SSTT). Three within-subjects factors were included: item type (trained, non-trained), training condition (feedback, no-feedback, restudy) and retention interval (10 min, 1 day, 1 week). Specifically, two groups of participants studied and trained once (ST) or twice (SSTT) the sentences. For each group, half of the items in these sentences were trained and the other half were not trained. During the training phase, the trained items were retrieved with feedback, without feedback or restudied. So, there were three training conditions for the trained items and the corresponding non-trained items. All the items in the sentences were then tested at three intervals.

**Figure 1:**
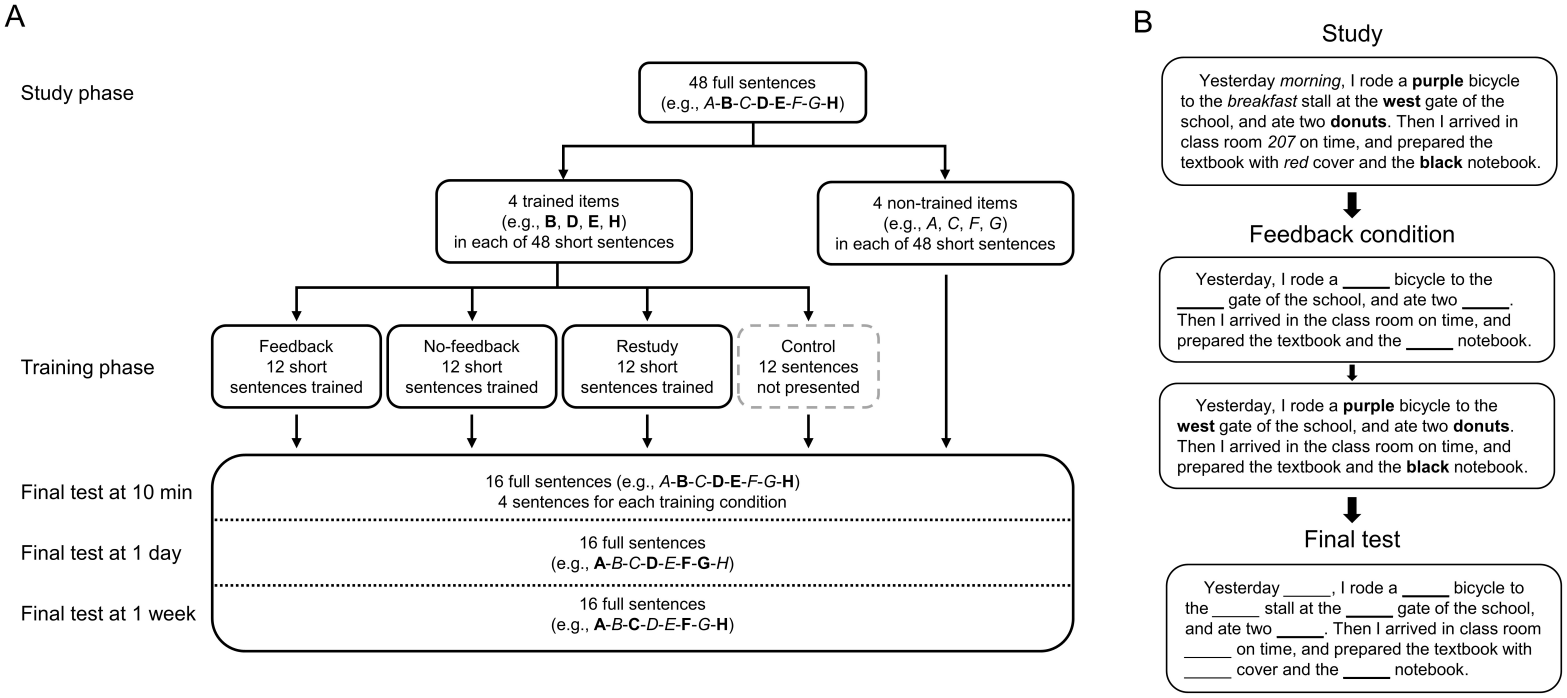
Diagram of materials. **(A)** Diagram of material sets. The items (e.g., item A-H) in each full sentence were randomly divided into four trained items (e.g., item B, D, E, H, in bold) and four non-trained items (e.g., item A, C, F, G, in italics). During the final tests, all the items were recalled, and the sentences at three intervals were different. **(B)** An example of sentences in different phases of the feedback condition. The sentences are translated into English and different item types are marked (e.g., trained items in bold, non-trained items in italics) for illustration purpose.

Forty-eight sentences that described different episodes of daily lives were used as materials (Wang and Yang, 2023). Each full sentence contained eight detailed items (e.g., items A-H), such as time, color, location, and quantity (Figure 1). To produce the trained items and non-trained items during the training phase, two corresponding short sentences with only half of the detailed items (e.g., items B, D, E, H; trained items) were created for each full sentence, whereas the other four items within the same sentence were not presented (e.g., items A, C, F, G; non-trained items) (Figures 1A, B). During the final test phase, both the four trained items and the four non-trained items (i.e., items A–H) were recalled in the full sentences (Figures 1A, B). There were 12 full sentences for each interval. The average length for the full sentences during study was 68.04 ± 3.57 Chinese characters (including punctuation marks), and the average length for the short sentences during training was 54.08 ± 3.52 characters (including punctuation marks). The mean logarithmic frequency (Friederic and Frisch, 2000) for the trained/non-trained item words was 9.37 ± 0.53, and the mean word length was 1.91 ± 0.83 characters. To control for the baseline level of the recall performance, 21 participants (4 males and 17 females, with mean age of 21.86 ± 2.52 years) who were not recruited in the study completed the sentences without undergoing a study phase. Here, the average baseline accuracy was 0.07 ± 0.07.

The sentences were randomly assigned into four sets as the materials for the training conditions of feedback, no-feedback, restudy and the control condition. Then the sentences in each set were randomly assigned into three subsets as the materials for three retention intervals (i.e., four sentences for each subset). Because each sentence had four trained items and four non-trained items, there were 16 items for each condition (e.g., trained in feedback condition at 10 min) (Figure 1A). The four sets and three subsets had no significant differences in their average baseline accuracy or lexical-semantic features, such as frequency, length and number of strokes for the detailed item words, as well as in the sentence length (*ps* > 0.500). The sets and subsets were counterbalanced, so that they had an equal opportunity of being used at different training conditions and retention intervals. The items were also counterbalanced, thus each item had an equal opportunity of being the trained and non-trained items.

### Procedure

**Figure 2:**
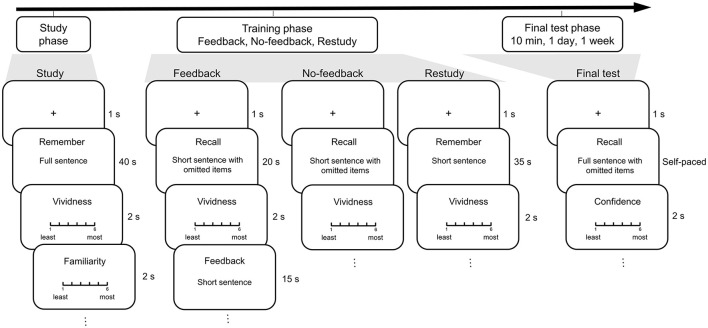
Procedure of the study, training and the final test phases. During the study phase, participants were presented with each of the full sentences that described episodes. During the training phase, the short sentences were trained in the blocks of feedback, no-feedback, and restudy conditions. During the final test phase, the participants were asked to recall the eight omitted items in the full sentences.

The experiment was conducted in a laboratory and consisted of three phases: study, training and final test. During the study phase, the participants studied all the full sentences (Figure 1B). They were presented with each of the 48 sentences for 40 s, during which they tried to remember the sentence by imagining the episodes described in the sentence (Figure 2). Then they judged to what extent they could imagine the episodes (1 as least vivid to 6 as most vivid) and to what extent they were familiar with the episodes (1 as least familiar to 6 as most familiar). The sentences were presented in a pseudo-random order so that no more than three sentences under the same condition were continuously presented. For the group ST, the participants studied the sentences once. For the group SSTT, the participants studied the sentences twice in two blocks with different random orders. After the study phase, they performed a distractor task of odd-even digit judgment for 2 min.

During the training phase, four conditions were included (Figure 1A). For the feedback condition, each of the 12 short sentences with only half of the information was presented for 20 s, among which four items were omitted. The participants were asked to recall the omitted items and rate the level of vividness (Figure 2). They spoke the items and their answers were recorded by the experimenter. Then the short sentence with only half of the information was provided as feedback for 15 s, among which the answers to the omitted items were listed (Figure 1B), and the participants remembered the short sentence again. For the no-feedback condition, each of the 12 short sentences with only half of the information was presented for 35 s, among which four items were omitted. The participants recalled the answers and rated the level of vividness, but no feedback was provided. For the restudy condition, each of the 12 short sentences with only half of the information was presented for 35 s, among which four items were listed. The participants remembered the short sentence and rated the vividness level. For the control condition, the 12 short sentences were not presented at all (Figure 1A).

The short sentences in the feedback, no-feedback and restudy conditions were presented in three blocks (Figure 2). The orders of the blocks and the trained/non-trained items in the sentences were counterbalanced across the participants. The sentences were presented in a pseudo-random order so that no more than three sentences at the same interval were continuously presented. The sentences were trained once for the group ST and twice in two blocks with different random orders for the group SSTT. After all the blocks, they performed distractor tasks including odd-even digit judgment and digital calculation (minus 7 from 1,000) for 10 min.

The participants performed final cued-recall tests at 10 min, 1 day and 1 week (Figure 2). During each test phase, each of the 16 full sentences was randomly presented with eight items omitted, four of which were trained while the other four were not. The participants recalled all the items and rated the confidence of their responses (1 as least sure to 6 as most sure). The sentences at the three intervals were different (Figure 1A). Before each of the formal phases, the participants had separate opportunities to practice trials.

### Data analysis

Memory accuracy was calculated as the proportion of correct answers out of the total items for each condition. The items under each training condition were divided into two types: trained and non-trained items under feedback, no-feedback, and restudy condition, respectively. To better identify the effect of answer feedback on memory over time, the testing effect, the transfer effect and the feedback effect were analyzed. Specifically, the testing effect was defined as the difference between the accuracy of feedback and restudy condition for the trained items, and the transfer effect was defined as the difference between the accuracy of feedback and restudy condition for the non-trained items. The feedback effect was defined as the difference between the accuracy of feedback and no-feedback condition for the trained items and non-trained items separately. For the items under the control condition, in which the sentences were learned but not presented at all during the training phase, their accuracy (ST: 0.39 ± 0.11; SSTT: 0.45 ± 0.15) was significantly lower than those under the training conditions (*ps* < 0.050). As we focused on memory difference between different training conditions, the control condition was not included in the analysis.

The data was first analyzed by a full factor ANOVA for memory accuracy, with item type (trained, non-trained), training condition (feedback, no-feedback, and restudy) and retention interval (10 min, 1 day, 1 week) as within-subjects factors and group (ST, SSTT) as a between-subjects factor. Then to disentangle the four-way interaction, separate ANVOAs were performed to identify how the factors of item type, interval and repetition modulated the memory effects. In these ANOVAs, item type (trained, non-trained), retention interval (10 min, 1 day, 1 week) and group (ST, SSTT) were treated as factors. The memory effects were also compared with chance level (0) to determine whether they significantly appeared in each condition. In addition, to examine the enhancement of repetition on the integration level, the ratings during the study and training phases in group SSTT were analyzed by paired *t*-tests with study repetition (first, second) or training repetition (first, second) as the factor. As an index of metacognition, confidence rating during the final tests was also analyzed by the AONVA with training condition (feedback, no-feedback, and restudy), retention interval (10 min, 1 day, 1 week) and group (ST, SSTT) as factors.

There were seven outliers (>2.5 SD) in the testing/transfer effect and feedback effect, which resulted in two participants in group ST and three participants in group SSTT having one or two outliers. When the outliers are excluded, all the data of the participant have to be excluded in the ANOVA analysis, which leads to smaller sample size and weaker analysis power. Thus, to ensure that sufficient number of participants were included in the ANOVA, the expectation maximization (EM) imputation was used to replace the missing values that were caused by the outliers (Schafer and Graham, 2002; Rashid and Gupta, 2021). Note that the results were similar when the participants with outliers were excluded from the corresponding analysis. Confidence ratings of 11 participants in group ST were not recorded due to program errors, resulting in confidence data of 61 participants to be analyzed.

The analyses were conducted in IBM SPSS Statistics 23. The effect sizes of the *F* and *t* statistics were reported using Partial Eta Squared ($\eta_{p}^{2}$) and Cohen's *d*, respectively. *Post-hoc* pairwise comparisons were Bonferroni-corrected (*p* < 0.050, two tailed). In addition, given the importance of the non-significant results associated with the null hypothesis (e.g., no significant transfer effect would be observed in group ST), Bayesian analyses were performed in JASP (Version 0.8.6) (JASP Team, 2018) to quantify the evidence in favor of the null effect. For the non-significant results in the ANOVA and one-sample *t*-test, Bayes factor (*BF*_01_) was reported, which was a ratio of the likelihood of the provided data given the null hypothesis to the likelihood of the data given the alternative hypothesis. Different from the null hypothesis significance testing (NHST), Bayes factor can indicate the extent to which the null hypothesis is more probable than the alternative hypothesis. The evidence in favor of the null hypothesis is moderate when *BF*_01_ is between 3 and 10 (Wagenmakers et al., 2018).

## Results

### Accuracy of the trained and non-trained items

**Figure 3:**
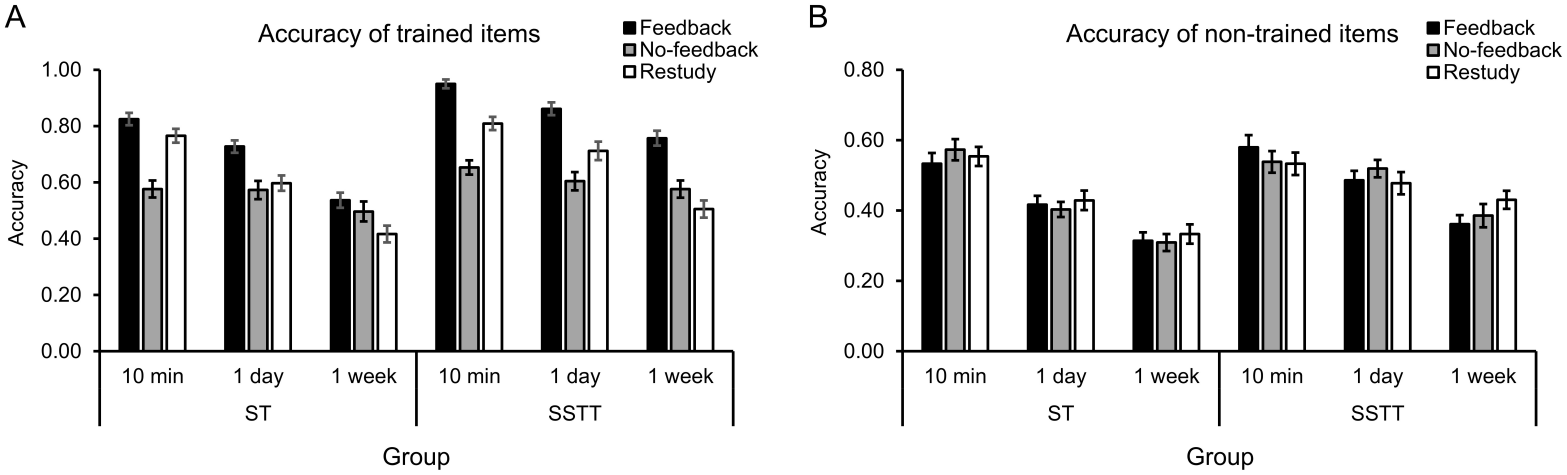
Results of recall accuracy in different conditions for the trained **(A)** and non-trained **(B)** items. The error bars represent the standard errors of means. ST, study once and training once; SSTT, study twice and training twice.

The full ANOVA for memory accuracy was first performed, with group as a between-subjects factor, and item type, training condition and retention interval as within-subjects factors. The main effects of group [*F*_(1, 70)_ = 8.39, *p* = 0.005, $\eta_{p}^{2}$ = 0.11], item type [*F*_(1, 70)_ = 513.68, *p* < 0.001, $\eta_{p}^{2}$ = 0.88], training condition [*F*_(2, 140)_ = 75.99, *p* < 0.001, $\eta_{p}^{2}$ = 0.52], and retention interval [*F*_(2, 140)_ = 246.26, *p* < 0.001, $\eta_{p}^{2}$ = 0.78] were significant. The interactions of group with other factors were significant [group ^*^ item type: *F*_(1, 70)_ = 7.87, *p* = 0.007, $\eta_{p}^{2}$ = 0.10; group ^*^ training condition: *F*_(2, 140)_ = 6.25, *p* = 0.003, $\eta_{p}^{2}$ = 0.08; group ^*^ interval: *F*_(2, 140)_ = 6.02, *p* = 0.003, $\eta_{p}^{2}$ = 0.08]. Further analysis showed that the accuracy of trained items was significantly higher in group SSTT than ST (*p* < 0.001) (Figure 3A), and the accuracy of non-trained items showed the same trend (*p* = 0.087) (Figure 3B). The accuracy was significantly higher in group SSTT than ST under feedback (*p* < 0.001) and restudy (*p* = 0.035) conditions, and this trend appeared under no-feedback condition (*p* = 0.056). In addition, the accuracy was significantly higher after SSTT at 1 day (*p* = 0.004) and 1 week (*p* < 0.001) than after ST, but not at 10 min (*p* = 0.165).

There was a significant interaction of item type ^*^ training condition [*F*_(2, 140)_ = 62.48, *p* < 0.001, $\eta_{p}^{2}$ = 0.47] and their interaction was modulated by group [*F*_(2, 140)_ = 3.28, *p* = 0.040, $\eta_{p}^{2}$ = 0.05] and interval [*F*_(4, 280)_ = 15.33, *p* < 0.001, $\eta_{p}^{2}$ = 0.18]. Further analysis showed that for the trained items (Figure 3A), the accuracy of feedback was the highest (*ps* < 0.001), and the accuracy of no-feedback was significantly lower than that of restudy only in group SSTT (*p* = 0.011) but not in ST (*p* = 0.120). Meanwhile, the accuracy was significantly lower under no-feedback than restudy condition at 10 min (*p* < 0.001) and 1 day (*p* = 0.018) but higher at 1 week (*p* = 0.012). For the non-trained items (Figure 3B), no significant differences were observed among training conditions regardless of group (*ps* > 0.500) and interval (*p* > 0.100). There was also a significant interaction of training condition ^*^ interval [*F*_(4, 280)_ = 6.31, *p* < 0.001, $\eta_{p}^{2}$ = 0.08], with the accuracy of feedback the highest at all intervals (*ps* < 0.020) and no significant difference between that of no-feedback and restudy at 1 day (*p* = 0.186) and 1 week (*p* = 0.575).

More importantly, the results showed a significant four-way interaction among group, item type, training condition and retention interval [*F*_(4, 280)_ = 4.00, *p* = 0.004, $\eta_{p}^{2}$ = 0.05]. To disentangle the interaction, the memory effects (i.e., memory difference between training conditions) were then analyzed in the following section. No other significant interactions were found (*ps* > 0.150, *BF*_01_ > 4.90).

### The testing effect and transfer effect (feedback vs. restudy)

**Figure 4:**
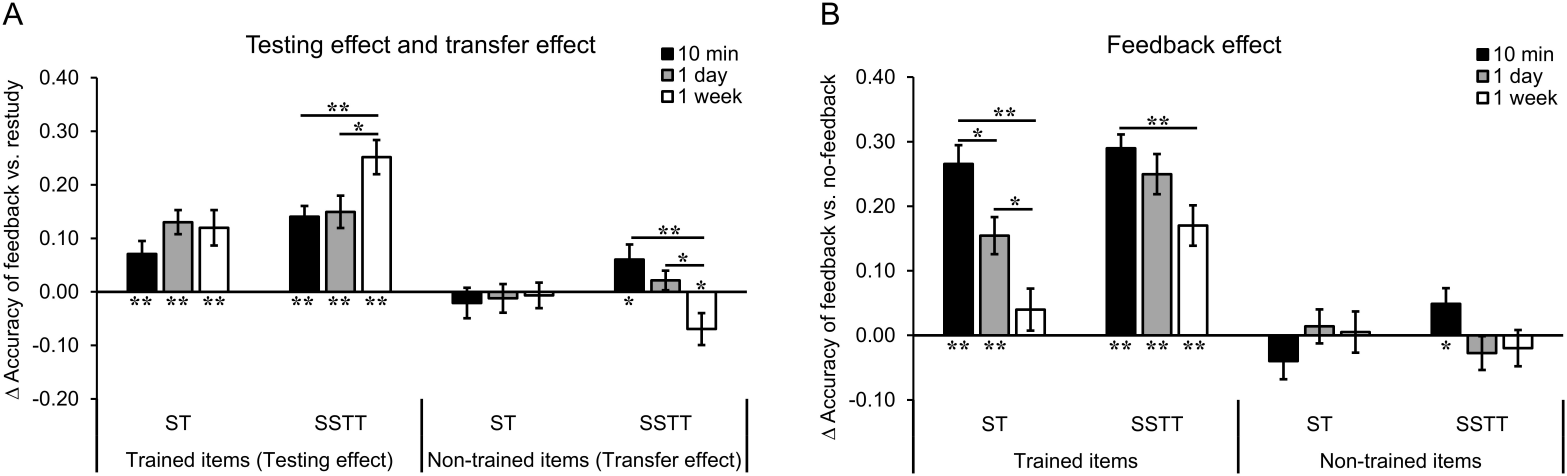
Results of the testing effect, transfer effect and the feedback effect. **(A)** The testing effect increased and the transfer effect decreased over time after SSTT but not after ST. The transfer effect significantly appeared at 10 min and was below the baseline at 1 week after SSTT. **(B)** The feedback effect for the trained items decreased over time after ST and SSTT. The feedback effect for the non-trained items appeared only at 10 min after SSTT. The error bars represent the standard errors of means. The asterisks above the bars represent significant difference between two intervals, and those below the bars represent significant difference from chance level (0) (**p < 0.01; *p < 0.05). Δ accuracy, accuracy difference; ST, study once and training once; SSTT, study twice and training twice.

To further clarify the extent to which memory effects were influenced by the factors of repetition, item type and retention interval, the accuracy difference between training conditions (i.e., feedback vs. restudy condition) was defined as the testing effect and transfer effect for the trained and non-trained items, respectively. Then the ANOVA with group (ST, SSTT), item type (trained, no trained) and retention interval (10 min, 1 day, 1 week) as factors was performed. The results showed significant main effects of group [*F*_(1, 70)_ = 10.95, *p* = 0.001, $\eta_{p}^{2}$ = 0.14] and item type [*F*_(1, 70)_ = 70.68, *p* < 0.001, $\eta_{p}^{2}$ = 0.50]. Consistent with our hypothesis, there was a significant two-way interaction of item type ^*^ interval [*F*_(2, 140)_ = 7.15, *p* = 0.001, $\eta_{p}^{2}$ = 0.09], as well as a significant three-way interaction of group ^*^ item type ^*^ interval [*F*_(2, 140)_ = 5.28, *p* = 0.006, $\eta_{p}^{2}$ = 0.07]. The three-way interaction indicated that the testing effect and transfer effect differed in the interaction of group and interval. Further analysis showed that the testing effect increased (*ps* < 0.045) and the transfer effect decreased significantly (*ps* < 0.050) from 10 min/1 day to 1 week only in group SSTT, while this pattern did not appear in group ST (*ps* > 0.450). Particularly, in group SSTT, the testing effect was significantly higher at 1 week than 10 min (*p* = 0.006) and 1 day (*p* = 0.042) (Figure 4A, left), and the transfer effect was significantly lower at 1 week than 10 min (*p* = 0.006) and 1 day (*p* = 0.049) (Figure 4A, right). The main effect of interval and other interactions were not significant (*ps* > 0.100, *BF*_01_ > 1.35). The results suggest that the factors of repetition and interval interactively influence the testing and transfer effects, with repeated study and training significantly improves the testing effect especially at longer interval, while repetition improves the transfer effect only at a short interval.

The difference of memory effects over chance level (0) could provide evidence whether memory effects are statistically reliable. When compared to baseline (0), the overall testing effect was significantly higher than 0 in group ST and SSTT (*ps* < 0.001) but the overall transfer effect was comparable to 0 in the two groups (*ps* > 0.200, *BF*_01_ > 2.65). The testing effect was significantly higher at each interval in both group ST and SSTT (*ps* < 0.010) (Figure 4A, left). The transfer effect was not significantly different from 0 at each interval in group ST (*ps* > 0.450, *BF*_01_ > 4.30), which was consistent with previous studies and our hypothesis. In group SSTT, the transfer effect was significantly higher than 0 at 10 min [*t*_(35)_ = 2.14, *p* = 0.039, *d* = 0.36], comparable to baseline at 1 day [*t*_(35)_ = 1.17, *p* = 0.250, *d* = 0.19, *BF*_01_ = 2.98] and significantly lower than 0 at 1 week [*t*_(35)_ = −2.32, *p* = 0.026, *d* = −0.39] (Figure 4A, right). Note that the effect sizes of the transfer effect at 10 min and 1 week in group SSTT were close to the weighted mean effect size (*d* = 0.40) in the meta-analysis of the transfer effect (Pan and Rickard, 2018), which confirmed that the transfer effect at 10 min and 1 week after SSTT was reliable. The results suggest that answer feedback promotes the testing effect irrespective of group and interval, but induces a reliably positive transfer effect only after repeated study and training, which limits to a shorter interval.

### The feedback effect (feedback vs. no-feedback)

In addition to the testing and transfer effects, the feedback effect was also analyzed to examine how the difference between feedback and no-feedback condition was influenced by various factors. The ANOVA with group (ST, SSTT), item type (trained, non-trained) and retention interval (10 min, 1 day, 1 week) as factors showed significant main effects of group [*F*_(1, 70)_ = 8.16, *p* = 0.006, $\eta_{p}^{2}$ = 0.10], item type [*F*_(1, 70)_ = 149.31, *p* < 0.001, $\eta_{p}^{2}$ = 0.68], and retention interval [*F*_(2, 140)_ = 7.95, *p* = 0.001, $\eta_{p}^{2}$ = 0.10]. There were significant two-way interactions of group ^*^ item type [*F*_(1, 70)_ = 5.32, *p* = 0.024, $\eta_{p}^{2}$ = 0.07] and item type ^*^ interval [*F*_(2, 140)_ = 11.42, *p* < 0.001, $\eta_{p}^{2}$ = 0.14]. Similar to the testing/transfer effect, there was a significant three-way interaction of group ^*^ item type ^*^ interval [*F*_(2, 140)_ = 6.48, *p* = 0.002, $\eta_{p}^{2}$ = 0.09]. Further analysis showed that for the trained items, the feedback effect decreased over time in group ST (*ps* < 0.030), while it decreased only from 10 min to 1 week in group SSTT (*p* = 0.008) (Figure 4B, left). For the non-trained items, the feedback effect did not change significantly over time in each group (*ps* > 0.150) (Figure 4B, right). In another direction, the feedback effect was enhanced by repetition at 1 day (*p* = 0.029) and 1 week (*p* = 0.005) but not at 10 min (*p* = 0.532) for the trained items, while was enhanced only at 10 min (*p* = 0.018) but not at 1 day (*p* = 0.274) and 1 week (*p* = 0.555) for the non-trained items. Other interactions were not significant (*ps* > 0.700, *BF*_01_ > 16.00). The results suggest that repeated study and training enhances the feedback effect for the trained items at long intervals, but for the non-trained items only at a shorter interval.

Similar to the testing and transfer effects, the feedback effect was compared to chance level (0) to examine whether it was statistically reliable. The results showed that the overall feedback effect was significantly higher than 0 for the trained items (*ps* < 0.001) but was comparable to 0 for the non-trained items in both groups (*ps* > 0.600, *BF*_01_ > 5.00). The feedback effect for the trained items was significantly higher at each interval (*ps* < 0.001) except at 1 week in group ST [*t*_(35)_ = 1.21, *p* = 0.236, *d* = 0.20, *BF*_01_ = 2.87] (Figure 4B, left). For the non-trained items, the feedback effect was not significantly different from 0 at each interval (*ps* > 0.150, *BF*_01_ > 2.15) except at 10 min in group SSTT [*t*_(35)_ = 2.03, *p* = 0.050, *d* = 0.34] (Figure 4B, right). The results suggest that providing answer feedback enhances memory of non-trained items at a shorter interval after repeated study and training when compared with no-feedback condition.

### Ratings during different phases

**Figure 5:**
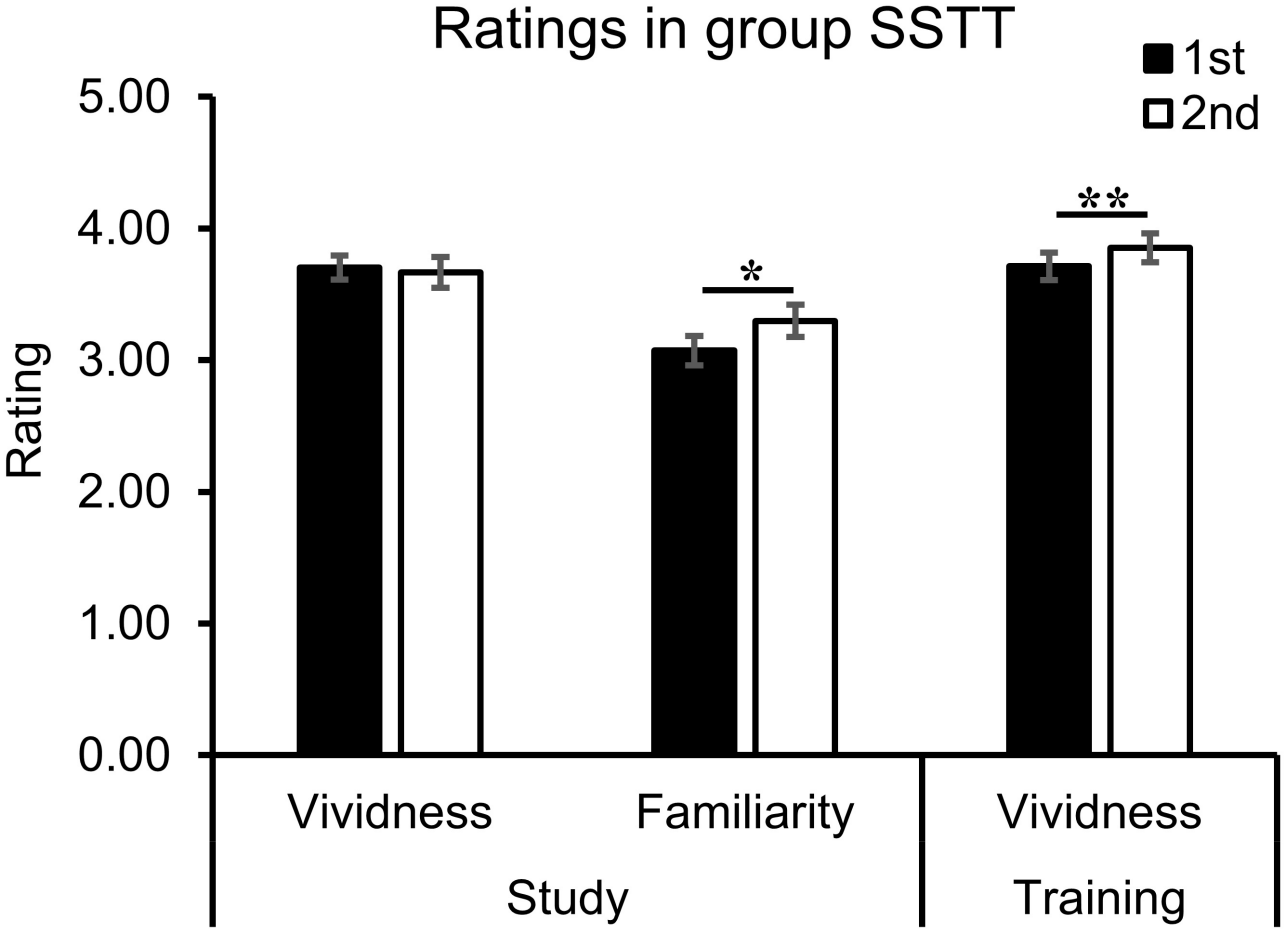
The ratings during study and training phases in group SSTT. Repeated study enhanced the level of familiarity, and repeated training enhanced the level of vividness. The error bars represent the standard errors of the means. The asterisks represent significant difference between two conditions (**p < 0.01; *p < 0.05). SSTT, study twice and training twice.

To explore the influence of repetition on the level of familiarity and vividness, the ratings during the study and training phases in group SSTT were analyzed with study repetition (first, second) or training repetition (first, second) as the factor. The results showed that repeated study significantly enhanced the level of familiarity [*t*_(35)_ = −2.59, *p* = 0.014, *d* = −0.43] (Figure 5, middle). The level of vividness significantly increased during training [*t*_(35)_ = −4.06, *p* < 0.001, *d* = −0.68], but remained stable during study [*t*_(35)_ = 0.49, *p* = 0.624, *d* = 0.08, *BF*_01_ = 4.98] (Figure 5, left and right).

To clarify how metacognition of memory changed as a function of various factors, the ANOVA with group, training condition and retention interval as factors was performed for the confidence rating during the final tests. The results showed significant main effects of group [*F*_(1, 59)_ = 7.00, *p* = 0.010, $\eta_{p}^{2}$ = 0.11], training condition [*F*_(2, 118)_ = 21.33, *p* < 0.001, $\eta_{p}^{2}$ = 0.27], and interval [*F*_(2, 118)_ = 119.27, *p* < 0.001, $\eta_{p}^{2}$ = 0.67]. There were significant interactions of training condition ^*^ group [*F*_(2, 118)_ = 3.14, *p* = 0.047, $\eta_{p}^{2}$ = 0.05] and training condition ^*^ interval [*F*_(4, 236)_ = 2.43, *p* = 0.049, $\eta_{p}^{2}$ = 0.04]. Further analysis showed that the confidence was significantly higher after SSTT than after ST in the feedback (*p* = 0.005) and restudy (*p* = 0.007) conditions and this trend appeared in the no-feedback condition (*p* = 0.071). In addition, the confidence decreased significantly over time in each training condition (*ps* < 0.005). Other interactions were not significant (*ps* > 0.100, *BF*_01_ > 1.70). The results suggest that repeated study and training not only improves memory accuracy, but also improves the confidence level for the answers.

## Discussion

In this study, the factors of group/repetition and retention interval were manipulated to explore the influence of answer feedback on memory for trained and non-trained episodic information over time. We found that the testing effect increased, but the transfer effect decreased over time only after SSTT but not after ST. Particularly, the transfer effect appeared at 10 min, but it decreased over time and was significantly below baseline at 1 week after SSTT. The feedback effect for the non-trained items also appeared at 10 min after SSTT. These results clarified the boundary conditions for the transfer of learning after answer feedback for episodic information, and highlighted the time change of memory specificity and generalization due to answer feedback.

### Answer feedback and the transfer effect

Although answer feedback significantly enhances memory of the trained items, its effect on memory of the non-trained items is not significant (vs. restudy) when semantic (e.g., Wooldridge et al., 2014; Tran et al., 2015; Pan et al., 2016a,b; Pan and Rickard, 2017) and episodic materials (e.g., Pickering et al., 2021) are adopted. Similarly, our results showed that an overall transfer effect was not significant in both groups. It broadly supports the view that the effect of answer feedback has small statistical power for the transfer of learning when compared to restudy condition (Pan and Rickard, 2018).

On the other hand, our study provided clear evidence that the non-significant transfer effect reflected the interaction between repetition and retention interval. There was a significant three-way interaction among item type, repetition and interval. That is, compared to restudy, answer feedback promoted memory of the non-trained items within the same episodic sentences only after SSTT at 10 min. Then the transfer effect decreased significantly over time and was significantly lower than baseline at 1 week. Furthermore, the effect size of significant interaction and that of the transfer effect at 10 min was close to those reported in previous studies (e.g., McDaniel et al., 2007; Eglington and Kang, 2018; Jonker et al., 2018) and in the meta-analysis (Pan and Rickard, 2018). The results suggest that with repeated study and training, the transfer effect due to answer feedback could be reliably obtained shortly after encoding of episodic information, but it becomes negative after long-term retention.

To our knowledge, this was the first study that found a significant transfer effect due to answer feedback for episodic memory. Unlike previous studies (e.g., Pickering et al., 2021), answer feedback produced a transfer effect when repeated study and repeated training were both applied. Previous studies have suggested that repeated training enhances information spreading and memory integration (Bjork et al., 2013; Jonker et al., 2018). To support this view, our results showed that the level of vividness increased significantly at the second training in group SSTT (see also Wang and Yang, 2023). Given that the transfer effect is absence when only repeated training is applied for episodic information (e.g., Pickering et al., 2021) and semantic materials (e.g., Wooldridge et al., 2014; Tran et al., 2015; Pan et al., 2016a,b; Pan and Rickard, 2017), repeated study seems necessary for the transfer effect. The three-factor framework for the transfer effect also proposes that when two factors (i.e., response congruency, elaborated retrieval) are absent, only if the initial performance exceeds a certain level is the transfer effect significant (Pan and Rickard, 2018). So applying both repeated study and repeated training is one of the boundary conditions to facilitate transfer of learning.

Moreover, the transfer effect decreased at a longer interval after SSTT. As shown in the meta-analysis of the transfer effect (Pan and Rickard, 2018), the trend of negative transfer effect due to answer feedback is usually observed after a few days of delay (e.g., 2 days−1 week. Pan et al., 2016b; Pickering et al., 2021). By including three intervals of 10 min, 1 day and 1 week, our results clarified that the transfer effect changed significantly over time with the interaction of repetition. That is, only after SSTT, rather than after ST, did the transfer effect decrease over time, leading to a negative transfer effect after 1 week. Thus, the transfer of learning due to answer feedback may not last for a long time even when repeated study and training is adopted. It also partly explains why previous studies found the negative transfer effect when only one interval (longer than 1 day) was applied (Pan and Rickard, 2018).

The underlying mechanism would be the balance of memory generalization and specificity changed by training conditions. Specifically, memory integration is enhanced during retrieval practice and feedback, so the non-trained items could be better recalled right after training. At the same time, feedback is fundamental to selectively strengthen the trained items and inhibit the non-trained items (Kornell et al., 2015; Kornell and Vaughn, 2016; Butler and Woodward, 2018), and the information spreading process persists over the passage of time due to retrieval practice and restudy (Carpenter, 2009; Karpicke et al., 2014; Pan and Rickard, 2018). The change in the two aspects leads to a decrease in the transfer effect due to answer feedback at 1 week.

### Transfer effect on episodic content

Our results showed that the transfer effect decreased but the testing effect increased over time after SSTT. Their different patterns help us better understand the nature of transfer of learning in our study. In addition to adopting episodic information, the critical manipulation of our study was that only half part of the sentences was trained (in three conditions including restudy), and the materials were different at three intervals. This ensured that the transfer effect was limited to information that was not exposed at all during the training phase but was related to the trained information, and the testing/transfer effects were not mixed with repetition effect by re-exposure of questions during the final test. Similar to our study, half of the materials were not presented during the training phase in some studies (e.g., questions in Chan et al., 2006; or scene-object associations in Jonker et al., 2018). The transfer effect in this type of studies is thus defined as the accuracy difference of items that are not presented during the training phase (for both feedback and restudy) in any format (Pickering et al., 2021), and belongs to the category of “untested materials seen during initial study” in Pan and Rickard (2018). Therefore, although the fill-in-the-blank retrieval practice was used (e.g., Hinze and Wiley, 2011; Pan et al., 2016a, 2019), we tested the transfer on content rather than the transfer on context/format.

Differently, in studies of the category of “stimulus-response rearrangement” (Pan and Rickard, 2018), the contents transferred in the final test are also presented during the training phase (e.g., study “gift, rose, wine,” train “gift, rose, ?” and final test “?, rose, win”) (e.g., McDaniel et al., 2007; Hinze and Wiley, 2011; Pan et al., 2016a,b, 2019; Pan and Rickard, 2017). Particularly, in some studies, triplets or sentences that are the same as those during encoding are presented in restudy condition (not part of them) (e.g., Pan et al., 2016a,b, 2019; Pan and Rickard, 2017). So, this type of transfer effect is defined as the difference between accuracy of test-difference and restudy (whole) condition, and reflects transfer of learning based on context/format (Carpenter, 2012). Another different situation is the transfer for the inference/application questions, which manifests as positive even when feedback is applied (e.g., Butler, 2010; McDaniel et al., 2012, 2015). In these studies, the answer to the inference/application question could be inferred from the feedback. In addition, when elaborative feedback (e.g., explanatory or detailed feedback) is provided, participants could rely on additional information to facilitate the transfer of learning. In these cases, repeated study may not be necessary, and the transfer effect could persist for a long time, which is similar to the testing effect (Roediger and Karpicke, 2006; Rowland, 2014). So how to manipulate the materials determines what type of transfer effect is tested, and what conditions are required for the reliable transfer of learning over time. Particularly, the transfer effect we explored and that in the category of “untested materials seen during initial study” is unique, which reflects more of test content rather than test context/format (Barnett and Ceci, 2002; Pan and Rickard, 2018).

### Feedback effect for trained and non-trained items

Our study also provided evidence for the role of feedback on memory of trained and non-trained items. The effect of feedback manifested two distinct features. First, memory performance was enhanced immediately after the training phase. The results were consistent with previous findings that feedback had a robust effect over short and long intervals for the trained items (Roediger and Butler, 2011; Rowland, 2014; Butler and Woodward, 2018; Carpenter et al., 2022). Particularly, the feedback effect was positive at 10 min after SSTT even for non-trained items. This highlighted the quick feature of feedback on memory. Second, the effect of feedback decreased over time for the trained items. Although providing the answer feedback can efficiently correct the errors, previous studies have suggested that initially incorrect items are more easily to be forgotten over time (Butler et al., 2011; Metcalfe and Miele, 2014; Wang and Yang, 2021). Differently, retrieval practice induces slower forgetting (e.g., Karpicke and Roediger, 2008). Note that participants attempt to retrieve the items before answer feedback is provided, so the feedback condition could be regarded as a combination of the retrieval practice and restudy for the trained items (Butler and Woodward, 2018). By repeated study and training, the feedback effect for the trained items remained significant at 1 week. The feedback is thus an efficient way to enhance memory retention of the trained information (Roediger and Butler, 2011; Rowland, 2014; Butler and Woodward, 2018) after SSTT.

### Theoretical and practical implications

This study has significant theoretical implications. First, the results clarified the boundary conditions for the effect of answer feedback on the transfer effect of episodic information. The significance of our study was that a positive transfer effect was observed at 10 min after SSTT. Compared with classroom studies (e.g., McDaniel et al., 2007, 2012), lab studies have the advantage to elucidate which factors are important for a robust transfer effect. Our results extend previous framework of the transfer effect (e.g., Pan and Rickard, 2018), and suggest that information integration and retention interval are important to interactively influence the transfer effect when answer feedback is provided. Although answer feedback is not a reliable moderator of transfer (Pan and Rickard, 2018), the transfer of learning after answer feedback is possible for episodic memory. Retrieving some information with answer feedback for a coherent episodic scenario could facilitate memory of non-trained information at a short interval when repeated study and training is adopted.

Second, the finding of transfer effect being both enhanced and inhibited reflects the flexibility of memory due to retrieval practice and answer feedback over time. The retrieval attempt facilitates information integration, boosts the recall of non-trained (but related) information and even revives forgotten memories (Kornell et al., 2015; Kornell and Vaughn, 2016; Bäuml and Trißl, 2022; Kriechbaum and Bäuml, 2023). On the other hand, repeated answer feedback has a strong selective function for the trained information, and the information without feedback is selectively inhibited. So at a longer interval, answer feedback led to weaker memory performance for non-trained items, and the transfer effect decreased after SSTT.

Therefore, our study highlights that the transfer of learning reflects the balance of generalization and specificity of memory. If generalization outweighs specificity, the transfer effect is observed, otherwise, the memory selectivity/specificity is observed. This is consistent with theories on feedback (e.g., Kornell and Vaughn, 2016), as well as the inhibition theory (e.g., Anderson, 2003) and the interference theory (e.g., Storm et al., 2015), which also emphasize the balance between memory of trained and non-trained information. This flexibility of memory also explains the significant transfer effect observed with elaborative feedback. As more information is provided by elaborative feedback, the new information could be integrated into previous representations, and acts as a cue for further retrieval (Pan and Rickard, 2018). Moreover, the content should be specific, but test format (e.g., from short-answer to multiple-choice) could be flexibly applied.

Our study also has significant practical implications. Students may not have enough time or opportunity to retrieve or restudy all the knowledge or episodic details that they have previously learned. Therefore, answer feedback still has the advantage to enhance non-trained information when only a short-term transfer effect is considered. On the other hand, if a long-lasting transfer effect is expected, additional effective strategies, such as retrieval practice/restudy and elaborative feedback (e.g., McDaniel et al., 2012, 2015; Pan et al., 2019) is necessary. At least restudy is an optimal strategy to enhance memory retention of non-trained semantic and episodic materials. More broadly, people could use different approaches to obtain specific outcomes in educational experiences. The feedback strategy is optimal for memory of trained information and memory specificity (Rowland, 2014; Butler and Woodward, 2018; Carpenter et al., 2022), whereas retrieval practice/restudy strategy is optimal for memory of non-trained information and memory generalization over time after repeated study and training.

## Limitation and future directions

This study has some limitations for future investigations. First, a within-subjects design was adopted for the training conditions in this study, by which the participants may use different strategies from those used in a between-subjects design (Yang et al., 2021; Carpenter et al., 2022). In addition, the materials we used were episodic sentences. Future studies could alternate the design and include semantic materials to confirm the time change of transfer effect after SSTT. Second, our study used an immediate answer feedback approach, and delayed feedback were not included. Previous studies have suggested that delayed feedback produces better memory retention than immediate feedback (Roediger and Butler, 2011; Rowland, 2014; Butler and Woodward, 2018). In addition to the type of feedback, other factors may interact with each other to influence the transfer effect, such as the correct proportion on the initial test (Pan and Rickard, 2018), prior knowledge (Pan et al., 2016a) and final test format (Tran et al., 2015; Pan et al., 2019). As transfer is a critically important goal in education (Carpenter, 2012; Pan and Rickard, 2018), to what extent these factors interact to rescue the long-term transfer effect requires further explorations.

## Conclusion

The question of how to improve the transfer of learning is of a great interest to both cognitive and learning sciences. The results showed that answer feedback promoted memory of the non-trained items for episodic information only after SSTT at 10 min. Moreover, the transfer effect due to answer feedback appeared immediately after the training, but it decreased over time and showed the inhibition of non-trained items at 1 week after SSTT. Differently, answer feedback promoted memory for the trained items irrespective of the number of study and training repetitions. The results clarified the boundary conditions for the transfer effect of answer feedback for episodic information over time, and highlighted the time change of memory specificity and generalization due to retrieval practice with answer feedback.

## Data Availability

The datasets presented in this study can be found in online repositories. The names of the repository/repositories and accession number(s) can be found below: https://osf.io/pn56a.

## References

[B1] Anderson M. C. (2003). Rethinking interference theory: executive control and the mechanisms of forgetting. J. Mem. Lang. 49, 415–445. 10.1016/j.jml.2003.08.006

[B2] Antony J. W. Ferreira C. S. Norman K. A. Wimber M. (2017). Retrieval as a fast route to memory consolidation. Trends Cogn. Sci. 21, 573–576. 10.1016/j.tics.2017.05.001 28583416 PMC5912918

[B3] Barnett S. M. Ceci S. J. (2002). When and where do we apply what we learn? A taxonomy for far transfer. Psychol. Bull. 128, 612–637. 10.1037/0033-2909.128.4.612 12081085

[B4] Bäuml K. T. Trißl L. (2022). Selective memory retrieval can revive forgotten memories. Proc. Natl. Acad. Sci. U. S. A. 119:e2114377119. 10.1073/pnas.2114377119 35165194 PMC8872727

[B5] Bjork E. L. Bjork R. A. MacLeod M. D. (2013). “Types and consequences of forgetting: Intended and unintended,” in Memory and Society: Psychological Perspectives, eds. Nilsson L. Ohta N. (London: Psychology Press), 134–158.

[B6] Butler A. C. (2010). Repeated testing produces superior transfer of learning relative to repeated studying. J. Exp. Psychol. Learn. Mem. Cogn. 36, 1118–1133. 10.1037/a0019902 20804289

[B7] Butler A. C. Fazio L. K. Marsh E. J. (2011). The hypercorrection effect persists over a week, but high-confidence errors return. Psychon. Bull. Rev. 18, 1238–1244. 10.3758/s13423-011-0173-y 21989771

[B8] Butler A. C. Karpicke J. D. Roediger H. L. (2008). Correcting a metacognitive error: feedback increases retention of low-confidence correct responses. J. Exp. Psychol. Learn. Mem. Cogn. 34, 918–928. 10.1037/0278-7393.34.4.918 18605878

[B9] Butler A. C. Woodward N. R. (2018). “Toward consilience in the use of task-level feedback to promote learning,” in Psychology of Learning and Motivation, ed. Federmeier K. D. (San Diego, CA: Elsevier Academic Press), 1–38. 10.1016/bs.plm.2018.09.001

[B10] Campbell J. I. D. Thompson V. A. (2012). MorePower 6.0 for ANOVA with relational confidence intervals and Bayesian analysis. Behav. Res. Methods 44, 1255–1265. 10.3758/s13428-012-0186-0 22437511

[B11] Carpenter S. K. (2009). Cue strength as a moderator of the testing effect: the benefits of elaborative retrieval. J. Exp. Psychol. Learn. Mem. Cogn. 35:1563. 10.1037/a0017021 19857026

[B12] Carpenter S. K. (2012). Testing enhances the transfer of learning. Curr. Dir. Psychol. Sci. 21, 279–283. 10.1177/0963721412452728

[B13] Carpenter S. K. Pan S. C. Butler A. C. (2022). The science of effective learning with spacing and retrieval practice. Nat. Rev. Psychol. 1, 496–511. 10.1038/s44159-022-00089-1

[B14] Chan J. C. K. (2009). When does retrieval induce forgetting and when does it induce facilitation? Implications for retrieval inhibition, testing effect, and text processing. J. Mem. Lang. 61, 153–170. 10.1016/j.jml.2009.04.004

[B15] Chan J. C. K. Mcdermott K. B. Roediger H. L. (2006). Retrieval-induced facilitation: initially nontested material can benefit from prior testing of related material. J. Exp. Psychol. Learn. Gen. 135, 553–571. 10.1037/0096-3445.135.4.553 17087573

[B16] Chua E. F. Schacter D. L. Sperling R. A. (2008). Neural correlates of metamemory: a comparison of feeling-of-knowing and retrospective confidence judgments. J. Cogn. Neurosci. 21, 1751–1765. 10.1162/jocn.2009.21123 18823230 PMC2709699

[B17] Dunlosky J. Metcalfe J. (2009). Metacognition. Thousand Oaks, CA: Sage.

[B18] Dunlosky J. Rawson K. A. Marsh E. J. Nathan M. J. Willingham D. T. (2013). Improving students' learning with effective learning techniques: promising directions from cognitive and educational psychology. Psychol. Sci. Public Interest 14, 4–58. 10.1177/1529100612453266 26173288

[B19] Eglington L. G. Kang S. H. K. (2018). Retrieval practice benefits deductive inference. Educ. Psychol. Rev. 30, 215–228. 10.1007/s10648-016-9386-y

[B20] Friederic A. D. Frisch S. (2000). Verb argument structure processing: the role of verb-specific and argument-specific information. J. Mem. Lang. 43, 476–507. 10.1006/jmla.2000.2709

[B21] Himmer L. Schonauer M. Heib D. P. J. Schabus M. Gais S. (2019). Rehearsal initiates systems memory consolidation, sleep makes it last. Sci. Adv. 5:eaav1695. 10.1126/sciadv.aav1695 31032406 PMC6482015

[B22] Hinze S. R. Wiley J. (2011). Testing the limits of testing effects using completion tests. Memory 19, 290–304. 10.1080/09658211.2011.560121 21500089

[B23] JASP Team (2018). JASP (Version 0.8.6). Available online at: https://jasp-stats.org/previous-versions/ (Accessed March 14, 2018).

[B24] Jonker T. R. Dimsdale-Zucker H. Ritchey M. Clarke A. Ranganath C. (2018). Neural reactivation in parietal cortex enhances memory for episodically linked information. Proc. Natl. Acad. Sci. U. S. A. 115, 11084–11089. 10.1073/pnas.1800006115 30297400 PMC6205442

[B25] Karpicke J. D. Lehman M. Aue W. R. (2014). “Retrieval-based learning: an episodic context account.” in Psychology of Learning and Motivation, Vol. 61, ed. Ross B. H. (San Diego, CA: Academic Press), 237–284. 10.1016/B978-0-12-800283-4.00007-1

[B26] Karpicke J. D. Roediger H. L. (2008). The critical importance of retrieval for learning. Science 319, 966–968. 10.1126/science.1152408 18276894

[B27] Kornell N. Hays M. J. Bjork R. A. (2009). Unsuccessful retrieval attempts enhance subsequent learning. J. Exp. Psychol. Learn. Mem. Cogn. 35, 989–998. 10.1037/a0015729 19586265

[B28] Kornell N. Klein P. J. Rawson K. A. (2015). Retrieval attempts enhance learning, but retrieval success (versus failure) does not matter. J. Exp. Psychol. Learn. Mem. Cogn. 41, 283–294. 10.1037/a0037850 25329079

[B29] Kornell N. Vaughn K. E. (2016). How retrieval attempts affect learning: a review and synthesis. Psychol. Learn. Motiv. 65, 183–215. 10.1016/bs.plm.2016.03.003

[B30] Kriechbaum V. M. Bäuml K. T. (2023). The critical importance of timing of retrieval practice for the fate of nonretrieved memories. Sci. Rep. 13:6128. 10.1038/s41598-023-32916-7 37061553 PMC10105692

[B31] Liu X. L. Ranganath C. (2021). Resurrected memories: sleep-dependent memory consolidation saves memories from competition induced by retrieval practice. Psychon. Bull. Rev. 28, 2035–2044. 10.3758/s13423-021-01953-6 34173188 PMC8642353

[B32] McDaniel M. A. Anderson J. L. Derbish M. H. Morrisette N. (2007). Testing the testing effect in the classroom. Eur. J. Cogn. Psychol. 19, 494–513. 10.1080/09541440701326154

[B33] McDaniel M. A. Bugg J. M. Liu Y. Brick J. (2015). When does the test-study-test sequence optimize learning and retention? J. Exp. Psychol. Appl. 21, 370–382. 10.1037/xap0000063 26501502

[B34] McDaniel M. A. Wildman K. M. Anderson J. L. (2012). Using quizzes to enhance summative-assessment performance in a web-based class: an experimental study. J. Appl. Res. Mem. Cogn. 1, 18–26. 10.1016/j.jarmac.2011.10.001

[B35] Metcalfe J. Miele D. B. (2014). Hypercorrection of high confidence errors: prior testing both enhances delayed performance and blocks the return of the errors. J. Appl. Res. Mem. Cogn. 3, 189–197. 10.1016/j.jarmac.2014.04.001

[B36] Pan S. C. Gopal A. Rickard T. C. (2016a). Testing with feedback yields potent, but piecewise, learning of history and biology facts. J. Educ. Psychol. 108, 563–575. 10.1037/edu0000074

[B37] Pan S. C. Hutter S. D'Andrea D. Unwalla D. Rickard T. C. (2019). In search of transfer following cued recall practice: the case of process-based biology concepts. Appl. Cogn. Psychol. 33, 629–645. 10.1002/acp.3506

[B38] Pan S. C. Rickard T. C. (2017). Does retrieval practice enhance learning and transfer relative to restudy for term-definition facts? J. Exp. Psychol. Appl. 23, 278–292. 10.1037/xap0000124 28358548

[B39] Pan S. C. Rickard T. C. (2018). Transfer of test-enhanced learning: meta-analytic review and synthesis. Psychol. Bull. 144, 710–756. 10.1037/bul0000151 29733621

[B40] Pan S. C. Wong C. M. Potter Z. E. Mejia J. Rickard T. C. (2016b). Does test-enhanced learning transfer for triple associates? Mem. Cogn. 44, 24–36. 10.3758/s13421-015-0547-x 26324093

[B41] Pashler H. Rohrer D. Cepeda N. J. Carpenter S. K. (2007). Enhancing learning and retarding forgetting: choices and consequences. Psychon. Bull. Rev. 14, 187–193. 10.3758/BF03194050 17694899

[B42] Pickering J. S. Henderson L. M. Horner A. J. (2021). Retrieval practice transfer effects for multielement event triplets. R. Soc. Open Sci. 8:201456. 10.1098/rsos.201456 34804558 PMC8580439

[B43] Pyc M. A. Rawson K. A. (2010). Why testing improves memory: mediator effectiveness hypothesis. Science 330, 335–335. 10.1126/science.1191465 20947756

[B44] Rashid W. Gupta M. K. (2021). “A perspective of missing value imputation approaches,” in Advances in Computational Intelligence and Communication Technology. Advances in Intelligent Systems and Computing, vol. 1086, eds. Gao X. Z. Tiwari S. Trivedi M. Mishra K. (Singapore: Springer Inc.), 307–315. 10.1007/978-981-15-1275-9_25

[B45] Roediger H. L. Butler A. C. (2011). The critical role of retrieval practice in long-term retention. Trends Cogn. Sci. 15, 20–27. 10.1016/j.tics.2010.09.003 20951630

[B46] Roediger H. L. Karpicke J. D. (2006). The power of testing memory: basic research and implications for educational practice. Perspect. Psychol. Sci. 1, 181–210. 10.1111/j.1745-6916.2006.00012.x 26151629

[B47] Rowland C. A. (2014). The effect of testing versus restudy on retention: a meta-analytic review of the testing effect. Psychol. Bull. 140, 1432–1463. 10.1037/a0037559 25150680

[B48] Schafer J. L. Graham J. W. (2002). Missing data: our view of the state of the art. Psychol. Methods 7, 147–177. 10.1037/1082-989X.7.2.147 12090408

[B49] Storm B. C. Angello G. Buchli D. R. Koppel R. H. Little J. L. Nestojko J. F. (2015). “A review of retrieval-induced forgetting in the contexts of learning, eyewitness memory, social cognition, autobiographical memory, and creative cognition.” in Psychology of Learning and Motivation, ed. Ross B. H. (Amsterdam: Elsevier Inc.), 141–194. 10.1016/bs.plm.2014.09.005

[B50] Tran R. Rohrer D. Pashler H. (2015). Retrieval practice: the lack of transfer to deductive inferences. Psychon. Bull. Rev. 22, 135–140. 10.3758/s13423-014-0646-x 24838305

[B51] Wagenmakers E. J. Love J. Marsman M. Jamil T. Ly A. Verhagen J.. (2018). Bayesian inference for psychology. Part II: Example applications with JASP. Psychon. Bull. Rev. 25, 58–76. 10.3758/s13423-017-1323-7 28685272 PMC5862926

[B52] Wang L. Yang J. (2021). Effect of feedback type on enhancing subsequent memory: interaction with initial correctness and confidence level. PsyCh J. 10, 751–766. 10.1002/pchj.481 34498410 PMC9293038

[B53] Wang L. Yang J. (2023). The influence of repeated study and repeated testing on the testing effect and the transfer effect over time. Mem. Cogn. 52, 476–490. 10.3758/s13421-023-01477-5 37874486

[B54] Wooldridge C. L. Bugg J. M. McDaniel M. A. Liu Y. (2014). The testing effect with authentic educational materials: a cautionary note. J. Appl. Res. Mem. Cogn. 3, 214–221. 10.1016/j.jarmac.2014.07.001

[B55] Yang C. Luo L. Vadillo M. A. Yu R. Shanks D. R. (2021). Testing (quizzing) boosts classroom learning: a systematic and meta-analytic review. Psychol. Bull. 147, 399–435. 10.1037/bul0000309 33683913

[B56] Yonelinas A. P. (1994). Receiver-operating characteristics in recognition memory: evidence for a dual-process model. J. Exp. Psychol. Learn. Mem. Cogn. 20, 1341–1354. 10.1037/0278-7393.20.6.1341 7983467

